# Contact behaviour before, during and after the COVID-19 pandemic in the Netherlands: evidence from contact surveys, 2016 to 2017 and 2020 to 2023

**DOI:** 10.2807/1560-7917.ES.2024.29.43.2400143

**Published:** 2024-10-24

**Authors:** Jantien A Backer, Eric R A Vos, Gerco den Hartog, Cheyenne C E van Hagen, Hester E de Melker, Fiona R M van der Klis, Jacco Wallinga

**Affiliations:** 1 National Institute for Public Health and the Environment (RIVM), Bilthoven, the Netherlands; 2 Leiden University Medical Center, Leiden, the Netherlands

**Keywords:** COVID-19, contact survey, contact behaviour, transmission potential, medical risk status, education level

## Abstract

**Background:**

The first wave of the COVID-19 pandemic in 2020 was largely mitigated by limiting contacts in the general population. In early 2022, most contact-reducing measures were lifted.

**Aim:**

To assess whether the population has reverted to pre-pandemic contact behaviour and how this would affect transmission potential of a newly emerging pathogen.

**Methods:**

We compared two studies on contact behaviour in the Netherlands: the PIENTER Corona study, conducted during and after the pandemic (held every 2–6 months from April 2020) and the PIENTER3 study (2016–17, as pre-pandemic baseline). In both, participants (ages 1–85 years) reported number and age group of all face-to-face persons contacted on the previous day in a survey. Transmission potential was examined using the next-generation matrix approach.

**Results:**

We found an average of 15.4 (95% CI: 14.3–16.4) community contacts per person per day after the pandemic in May 2023, 13% lower than baseline (17.8; 95% CI: 17.0–18.5). Among all ages, children (5–9 years) had the highest number of contacts, both pre- and post-pandemic. Mainly adults aged 20–59 years had not reverted to pre-pandemic behaviours, possibly because they more often work from home. Although the number of contacts is lower compared to the pre-pandemic period, the effect on transmission potential of a newly emerging respiratory pathogen is limited if all age groups were equally susceptible.

**Conclusion:**

Continuous monitoring of contacts can signal changes in contact patterns and can define a ‘new normal’ baseline. Both aspects are needed to prepare for a future pandemic.

**Key public health message**

**What did you want to address in this study and why?**

During the COVID-19 pandemic, many individuals reduced the number of contacts with people outside their own household to slow virus transmission. Given that contact-reducing measures were lifted in early 2022, we would like to understand whether contact behaviour is similar to before the pandemic and, if not, how that would affect a future pandemic. We compare pre- and post-pandemic contact behaviour in the Netherlands using contact surveys.

**What have we learnt from this study?**

Children and young adults aged under 20 years had the highest number of contacts (around 25) per person per day, similar to before the pandemic. Adults aged 20–59 years had a lower number of contacts per person per day after the pandemic vs before (19 vs 14), possibly because they work more often from home.

**What are the implications of your findings for public health?**

Despite the changed contact behaviour, a new respiratory pathogen could still spread in a completely susceptible population as easily as before the pandemic, as the epidemic is driven by the 0–20-year-olds who have the highest number of contacts. Should they be less susceptible to infection by a new pathogen, transmission potential would be lower and a future outbreak could be controlled with less effort than before the COVID-19 pandemic.

## Introduction

After the severe acute respiratory syndrome coronavirus 2 (SARS-CoV-2) emerged at the end of 2019, the World Health Organization (WHO) declared the outbreak a global pandemic on 11 March 2020 [1]. Most nations implemented stringent non-pharmaceutical interventions to mitigate virus spread, including physical distancing aimed at reducing exposure to SARS-CoV-2 by lowering contact rates. Face-to-face and physical contacts between members of different households were to be reduced, as each contact was considered an at-risk event for transmission. Many contact surveys that were conducted during the COVID-19 pandemic showed that more stringent measures indeed led to lower contact rates [2-10].

During the COVID-19 pandemic, the change in behaviour varied widely among groups in the population, either because they were affected by control measures differently or because they differed in ability or willingness to comply. For instance, the number of contacts in younger age groups were greatly impacted by school closures [11], while the number of contacts in older age groups decreased with suspended social gatherings [8,12]. Persons with comorbidities may have reduced their contacts more than those without comorbidities [8,13,14], given their higher probability of severe outcomes [15]. Also, the socioeconomic status of a person may have influenced contact behaviour, as those with a low socioeconomic status often have professions that preclude working from home [16].

Most contact-reducing measures were lifted shortly after the emergence of the more transmissible SARS-CoV-2 Omicron variants in late 2021. It is important to know whether contacts in the population reverted to their pre-pandemic levels because a lasting change in contact behaviour might affect a future pandemic situation. Answering these questions would require a comparison of the contact behaviour in post-pandemic period, including all age groups, with a valid baseline measurement before the pandemic started. In the Netherlands, two contact behaviour studies were conducted as part of a larger serosurveillance study encompassing a large nationwide sample of the Dutch population. The PIENTER Corona [17] study began in April 2020 and will conclude at the end of 2024, and includes contact survey rounds every 2–6 months. The study’s design closely mirrors that of a study conducted in 2016–17 (PIENTER3 [18]), which provides a pre-pandemic baseline of contact behaviour. An earlier analysis revealed a reduction of 76% and 41% in the number of contacts outside households during the April 2020 and June 2020 survey rounds, respectively, in comparison to the baseline survey [19].

On 25 February 2022, all contact-reducing measures were lifted in the Netherlands. Using these two data sets, we aimed to compare the contact behaviour in the PIENTER Corona survey rounds before and after this date to the contact behaviour before the pandemic. We studied the differences in contacts between age groups, medical risk groups and socioeconomic groups, and we assessed the potential implications for a future outbreak of a respiratory pathogen.

## Methods

### Study design and population

The PIENTER3 study, referred to as ‘the baseline study’, was conducted from February 2016 to October 2017 in a representative sample of the Dutch population [18], who were randomly selected from the national Personal Records Database (BRP) [20]. Of these participants, 80% were invited for the first round of the PIENTER Corona (referred to as PiCo) survey conducted in April 2020 [17], and they were reinvited every 2–6 months for in total 10 survey rounds until May 2023. 

The study population was supplemented with a random selection from persons in the BRP older than 1 year in PiCo survey rounds 2 (June 2020) [21] and 6 (November 2021) [22], who were also reinvited for each subsequent round. For comparability, the baseline participants younger than 1 year old were excluded from the analysis.

### Survey questionnaire

For each round, questionnaires were filled out online or on paper either by the participant or with help from their parent or guardian if they were younger than 15 years old. Participants reported their age and sex, as well as the age and sex of their household members. From a list of self-reported medical conditions, we determined whether participants would be eligible for influenza vaccination according to the national guidelines [23]. Participants with an indication for influenza vaccination were classified as having a high medical risk status.

Participants provided their highest obtained or current education level, which served as a proxy for socioeconomic status. The education level was classified according to Dutch standards [24] as low (no education or primary education), medium (secondary school or vocational training) or high (bachelor’s degree, university). Participants up to 14 years old were assigned the highest education level of their parents or guardians. Participants were also asked whether they worked from home in the previous week.

The questionnaire included a section on contact behaviour, where contacts were defined as unique persons with whom the participant talked face-to-face, touched, kissed or played sports. Participants listed the number of contacts they had with persons outside their household on the previous day. The contacts were stratified by age group and either by sex (male/female) or by proximity (less/more than 1.5 m apart). Participants who did not provide valid contact data were excluded from the analysis.

A detailed description on the participant selection and participation, the definition of the medical risk status, the validity of contact data and handling of changes in survey questions is provided in Supplementary Material S1. The data were reformatted to the standard format for contact surveys on socialcontactdata.org and published online [25]. The code for data cleaning and the analysis are available online [26].

### Contact analysis

The age groups of participants and contacts were aggregated in 10 age groups: 0–4, 5–9, 10–19, 20–29, 30–39, 40–49, 50–59, 60–69, 70–79 and ≥ 80 years. In each round, the number of community contacts per participant per contact age group was truncated at 50, i.e. a maximum of 500 community contacts per participant. The household composition of the participant was used as a proxy for contacts within the household, assuming the participant would have contacted each household member on the survey day. The community and household contacts were analysed separately.

Within each participant age group, participants were weighted according to the age and sex distribution of the Dutch population [27]. Participants were also weighted according to weekday (weight 5/7) or weekend (weight 2/7), but only for community contacts, assuming the household composition is constant over the week. With these sample weights, the weighted mean number of contacts per participant in that participant age group and contact age group was calculated. These numbers are the elements of a contact matrix. Assuming all contacts are reciprocal, we corrected the contact matrix for reporting errors using the age distribution of the Dutch population [28]. The confidence interval was expressed by the 95% bias-corrected bootstrap interval [29], based on 1,000 bootstrap samples by participant age group.

The number of community contacts per participant per day was compared with the baseline values that were obtained from the 2016–17 contact survey. We studied the number of contacts over time and by age group. The reported contacts by medical risk status and education level were expected to be confounded by the age of the participants. To be able to compare the subgroups, we weighted their age groups by the age distribution of the general population and calculated the population average. Such a population average of e.g. the high medical risk status group should therefore be interpreted as the population average if the entire population would have a high medical risk status.

### Analysis of next-generation matrix

A total contact matrix was constructed by summing the contact matrices of the community and household contacts. If all age groups were equally infectious and susceptible to a newly emerging pathogen, this matrix can be interpreted as the next-generation matrix (NGM) of an epidemic process [30]. In a fully susceptible population without any control measures, the spectral radius, i.e. maximum eigenvalue, of the NGM is proportional to the basic reproduction number R_0_, indicating the average number of secondary cases infected by a typical primary case [31]. The spectral radius of the NGM can therefore be interpreted as a proxy for the transmission potential of a newly emerging pathogen for which no prior immunity exists. We compared the spectral radius over time to the baseline value. The rounds without any control measures, i.e. after survey round 7, reflect the transmission potential of a newly emerging pathogen under ‘new normal’ circumstances compared with the pre-COVID-19 period. When age groups differ in susceptibility to infection or infectiousness after infection, the NGM changes accordingly. In the early phase of the pandemic, Zhang et al. [2], found that 0–14-year-olds are 2.9 times less susceptible than adults aged 15–64 years and that adults aged 65 years and older are 47% more susceptible than adults aged 15–64 years. We used these values to determine the transmission potential of a newly emerging pathogen that would resemble the early SARS-CoV-2 virus.

## Results

Our final dataset comprises 62,490 questionnaires, completed by 13,826 unique participants, 97% of which also provided their household composition (n = 13,154). The number of participants in the PiCo survey ranged from 2,594 in the first round when only baseline participants were reinvited to 8,144 in the sixth round when the study population was supplemented (Table). In total, 5,768 persons participated in the baseline survey (survey round 0 in Table), 2,487 of whom also participated in one or more (on average 6.6) rounds of the PiCo survey. The participants recruited in survey rounds 2 and 6 participated in on average 6.1 and 3.4 rounds, respectively. From the baseline study, 387 participants younger than 1 year were excluded for the analysis; from the baseline and PiCo studies, 1,780 questionnaires without valid contact data had already been excluded.

**Table:** Characteristics of the study population, by survey month, the Netherlands, 2016–2017 (n = 5,381) and 2020–2023 (n = 56,722)

Survey month	Survey round																						
	0		1		2		3		4		5		6		7		8		9		10		Ref^c^
	Baseline^a^ (n = 5,381)		Apr 2020 (n = 2,594)		Jun 2020 (n = 6,704)		Oct 2020 (n = 6,086)		Mar 2021 (n = 5,912)		Jul 2021 (n = 5,231)		Nov 2021 (n = 8,144)		Apr 2022 (n = 6,347)		Jul 2022 (n = 5,626)		Nov 2022 (n = 5,248)		May 2023 (n = 4,830)		
	n	%	n	%	n	%	n	%	n	%	n	%	n	%	n	%	n	%	n	%	n	%	
Participant age group (years)																							
0–4	341	6.3	189	7.3	227	3.4	154	2.5	105	1.8	46	0.9	219	2.7	116	1.8	87	1.5	56	1.1	46	1.0	4.9
5–9	349	6.5	129	5.0	280	4.2	224	3.7	193	3.3	167	3.2	411	5.0	263	4.1	201	3.6	143	2.7	113	2.3	5.2
10–19	624	11.6	224	8.6	605	9.0	503	8.3	472	8.0	363	6.9	652	8.0	483	7.6	374	6.6	322	6.1	288	6.0	11.4
20–29	836	15.5	315	12.1	689	10.3	558	9.2	552	9.3	414	7.9	769	9.4	510	8.0	394	7.0	339	6.5	291	6.0	12.9
30–39	748	13.9	378	14.6	762	11.4	695	11.4	706	11.9	576	11.0	887	10.9	658	10.4	536	9.5	470	9.0	421	8.7	12.4
40–49	663	12.3	351	13.5	904	13.5	816	13.4	801	13.5	730	14.0	966	11.9	750	11.8	637	11.3	615	11.7	552	11.4	12.6
50–59	620	11.5	400	15.4	1,089	16.2	1,024	16.8	1,013	17.1	932	17.8	1,108	13.6	907	14.3	831	14.8	801	15.3	746	15.4	14.6
60–69	686	12.7	330	12.7	1,198	17.9	1,150	18.9	1,109	18.8	1,038	19.8	1,151	14.1	958	15.1	927	16.5	897	17.1	841	17.4	12.2
70–79	420	7.8	234	9.0	812	12.1	815	13.4	815	13.8	813	15.5	1,386	17.0	1,210	19.1	1,139	20.2	1,120	21.3	1,036	21.4	9.1
≥ 80	94	1.7	44	1.7	138	2.1	147	2.4	146	2.5	152	2.9	595	7.3	492	7.8	500	8.9	485	9.2	496	10.3	4.7
Participant sex																							
Female	2,980	55.4	1,430	55.1	3,709	55.3	3,397	55.8	3,323	56.2	2,967	56.7	4,609	56.6	3,591	56.6	3,174	56.4	2,952	56.2	2,720	56.3	50.3
Male	2,401	44.6	1,164	44.9	2,995	44.7	2,689	44.2	2,589	43.8	2,264	43.3	3,535	43.4	2,756	43.4	2,452	43.6	2,296	43.8	2,110	43.7	49.7
Household size																							
1	748	13.9	198	7.6	700	10.4	666	10.9	647	10.9	616	11.8	1,060	13.0	871	13.7	802	14.3	767	14.6	725	15.0	18.1
2	1,866	34.7	808	31.1	2,578	38.5	2,491	40.9	2,464	41.7	2,293	43.8	3,391	41.6	2,817	44.4	2,639	46.9	2,520	48.0	2,334	48.3	30.7
3	687	12.8	420	16.2	972	14.5	860	14.1	820	13.9	693	13.2	987	12.1	726	11.4	601	10.7	566	10.8	524	10.8	16.6
4	1,067	19.8	711	27.4	1,609	24.0	1,372	22.5	1,319	22.3	1,116	21.3	1,755	21.5	1,281	20.2	1,009	17.9	919	17.5	812	16.8	22.6
≥ 5	629	11.7	331	12.8	818	12.2	695	11.4	639	10.8	511	9.8	922	11.3	645	10.2	513	9.1	468	8.9	411	8.5	12.0
Missing	384	7.1	126	4.9	27	0.4	2	0.0	23	0.4	2	0.0	29	0.4	7	0.1	62	1.1	8	0.2	24	0.5	NA
Medical risk status^b^																							
Low	3,290	61.1	1,796	69.2	4,547	67.8	3,795	62.4	4,476	75.7	3,956	75.6	5,868	72.1	4,607	72.6	4,045	71.9	3,646	69.5	3,273	67.8	78.6
High	1,399	26.0	797	30.7	2,155	32.1	969	15.9	1,436	24.3	1,275	24.4	2,276	27.9	1,683	26.5	1,581	28.1	1,555	29.6	1,512	31.3	21.4
Missing	692	12.9	1	0.0	2	0.0	1,322	21.7	0	0.0	0	0.0	0	0.0	57	0.9	0	0.0	47	0.9	45	0.9	NA
Education level																							
Low	1,292	24.0	420	16.2	1,262	18.8	1,160	19.1	1,094	18.5	1,003	19.2	1,538	18.9	1,207	19.0	1,129	20.1	1,076	20.5	1,030	21.3	28.6
Medium	1,731	32.2	805	31.0	2,154	32.1	1,988	32.7	1,924	32.5	1,716	32.8	2,384	29.3	1,866	29.4	1,663	29.6	1,571	29.9	1,471	30.5	37.8
High	2,093	38.9	1,258	48.5	3,256	48.6	2,928	48.1	2,860	48.4	2,499	47.8	4,209	51.7	3,241	51.1	2,807	49.9	2,601	49.6	2,329	48.2	33.6
Missing	265	4.9	111	4.3	32	0.5	10	0.2	34	0.6	13	0.2	13	0.2	33	0.5	27	0.5	0	0.0	0	0.0	NA

NA: not applicable.

^a^ The baseline survey from 2016 to 2017 is indicated as survey round 0. The n at round 0 is the number of participants (n = 5,768) minus the under 1-year-olds (n = 387).

^b^ The medical risk status of participants was classified as high if they had an indication for influenza vaccination, based on self-reported medical conditions, or low otherwise. The education level of participants was classified as low (no education or primary education), medium (secondary school or vocational training) or high (bachelor’s degree, university).

^c^ The final column contains reference percentages for the general population by age group [27], sex [27], household size [32], medical risk status [33] and education level for 15–90-year-olds [24].

**Figure 1:**
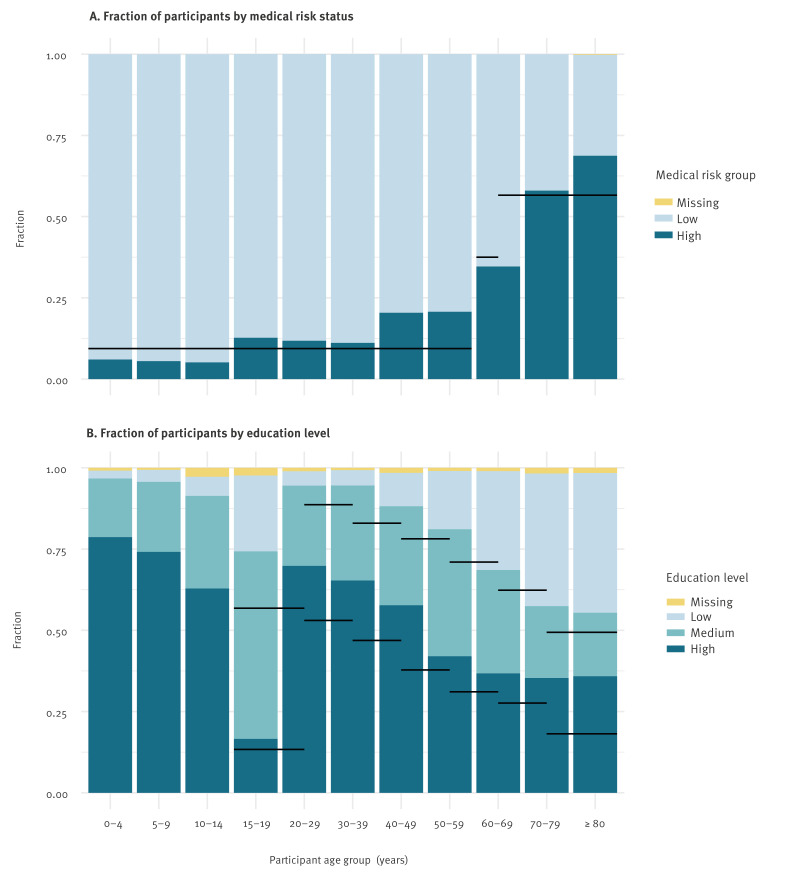
Medical risk status and education level of participants by age group, the Netherlands, April 2020–November 2022 (n = 8,211)

Generally, age groups under 40 years were underrepresented, and age groups between 60 and 80 years were overrepresented compared with the Dutch population [27]. This imbalance seemed to increase over time, suggesting a higher drop-out rate for younger age groups. The study population contained on average 56% female participants, a percentage which was constant over all rounds. Participants living in two-person households were overrepresented [32]. This was partly explained by the higher age of the participants; when the household size frequency was corrected for confounding by age and sex, two-person households were less overrepresented, but single-person households remained underrepresented. Supplementary Table S2 provides the characteristics of the study population corrected for confounding, using the age group and sex of the general population. The fraction of participants with a high medical risk was higher than in the general population [33] (Figure 1A), but this was completely explained by confounding with age and sex. The education level of the participants was higher than in the general population [24] (Figure 1B). This overrepresentation became even more pronounced after correcting for age and sex confounding, because the abundant older participants had on average a lower education level.

A. Fraction of participants by medical risk status, for unique participants from PIENTER Corona (PiCo) survey round 4 (March 2021) onwards (n = 3,344). From this round onwards, participants were asked to report current medical conditions, before this round also past medical conditions were reported. Reference values (black lines) are taken from [33].

B. Fraction of participants by education level, for unique participants. For participants aged under 15 years, the highest education level of the parents or guardians is used. For comparability, the age group 10–19 years is split into 10–14 and 15–19 years. Reference values (black lines) are taken from [24].

### Number of community contacts

**Figure 2:**
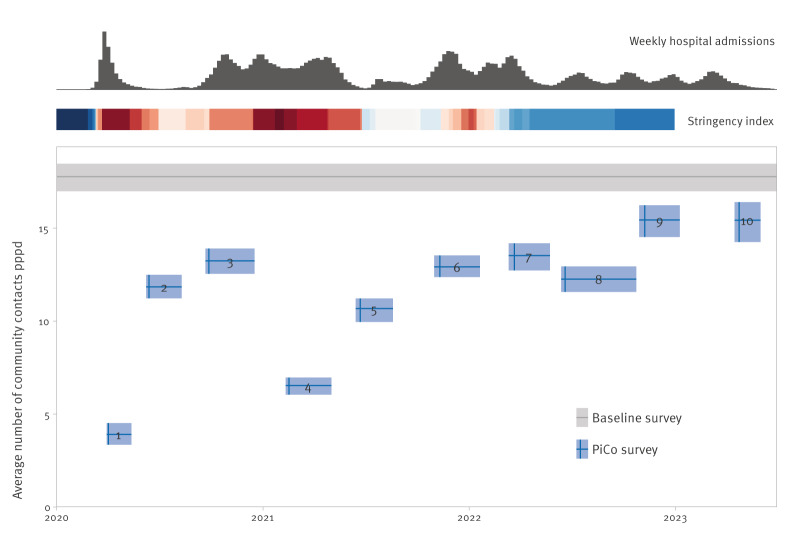
Weighted average number of community contacts per person per day in the general population for the baseline and PiCo studies, the Netherlands, April 2020–November 2022 (n = 62,103)

PiCo: PIENTER Corona study; pppd: per person per day.

The number of community contacts in the general population varied over the course of the COVID-19 pandemic (Figure 2). Numbers were low in periods with high numbers of hospital admissions and stringent measures, such as school closures and restrictions on mass gatherings. Around the start of 2022 when the Omicron variant became dominant, a short period of lockdown measures did not seem to affect the average number of contacts to the same extent, because 99% of questionnaires in survey round 6 were filled out before these measures came into effect on 19 December 2021. Since physical distancing measures were later lifted on 25 February 2022, the number of community contacts increased from 13.5 (95% CI: 12.7–14.2) community contacts per person per day in survey round 7 to 15.4 (14.3–16.4) in survey round 10. However, these latest numbers were still 13% lower than the baseline value of 17.8 (17.0–18.5) contacts per day.

Weighted average number of contacts (horizontal line) and 95% confidence interval (shaded area) are indicated. PiCo rounds are shown from the start to end date, with the median survey date (vertical line) with the survey round number in black. The baseline survey was conducted in the period from February 2016 to October 2017.

As a timeline reference, the weekly number of hospital admissions [40] and the Oxford Stringency Index [41] are depicted on top. The stringency index is colour coded from blue (low) to red (high).

For 1,262 of 62,103 questionnaires, the number of community contacts was truncated as they exceeded the maximum of 50 contacts per contact age group. Because of this truncation the 95th percentile of the number of community contacts per participant decreased from 59 to 54.

**Figure 3:**
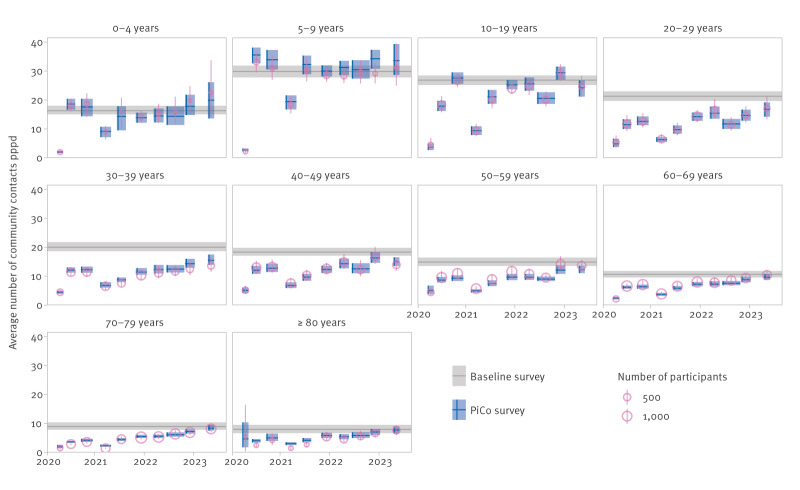
Modelled average number of community contacts per participant per day in the general population by age group for the baseline and PiCo studies, the Netherlands, April 2020–November 2022 (n = 62,103)

PiCo: PIENTER Corona study; pppd: per person per day.

When examining the results in more detail, we observed large differences between age groups (Figure 3). In the baseline survey, the number of contacts was highest for the 5–9-year age group and gradually decreased with age. During the pandemic, the number of contacts of the age groups under 10 years was around the baseline value, with the exception of the lockdown periods (survey rounds 1 and 4) that included primary school and daycare closures. Compared with this group, the contacts of 10–19-year-olds showed more variation, but they have reverted to pre-pandemic levels from 2022 onwards. For all other age groups, the number of contacts were well below baseline during the first 2 pandemic years. In the last survey rounds 9 and 10, the age groups of 60 years and older approached pre-pandemic behaviour, but the 20–59-year-olds have maintained a below-baseline level.

The average number of contacts (horizontal line) and 95% confidence interval (shaded area) are indicated. PiCo rounds are shown from the start to end date, with the median survey date (vertical line). For the PiCo rounds, the reported number of contacts is shown (open circle) with the 95% confidence interval (whisker) where the size of the circle is scaled by the number of participants. The baseline survey was conducted in the period from February 2016 to October 2017. Reciprocity of contacts is assumed for the modelled number of contacts, not for the reported number of contacts.

The difference between the modelled (rectangles, Figure 3) and reported (circles, Figure 3) number of contacts reflect how much the reported contacts needed to be adjusted to comply with the assumption of reciprocal contacts, i.e. with perfect reporting behaviour, the rectangles and circles would coincide. However, for the 5–9-year-olds, for instance, the modelled number of contacts is higher than the reported number of contacts in all survey rounds. This indicates an inconsistency where 5–9-year-olds reported fewer contacts with other age groups than other age groups with them. Generally, differences between modelled and reported numbers of contacts were smaller than the uncertainty, indicating consistent reporting behaviour.

**Figure 4:**
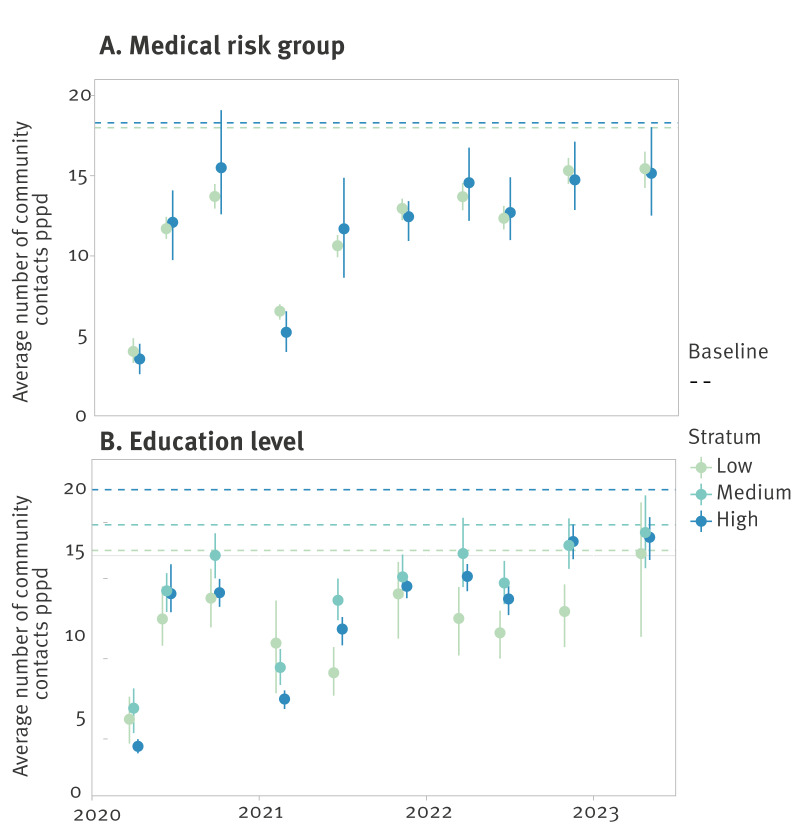
Weighted average number of community contacts per person per day by medical risk status (n = 59,937) and education level (n = 61,565) for the baseline and PiCo studies, the Netherlands, April 2020–November 2022

PiCo: PIENTER Corona study; pppd: per person per day.

In the baseline survey, the average number of community contacts did not differ by medical status group (Figure 4). Similarly, in the PiCo survey rounds, no difference between the two medical risk groups was observed. However, in the baseline survey, the average number of community contacts increased with education level (Figure 4). In the PiCo rounds with the most stringent measures (survey rounds 1 and 4), this ranking was reversed. In all other rounds with less stringent measures, the average number of community contacts for the low education level is lowest, while middle and high education levels have a similar higher number of contacts.

The averages are calculated by weighting the age groups in each stratum by the age distribution of the general population and can be interpreted as the number of contacts if the full population consisted of a single stratum. Shown is the average number of contacts (point) and 95% confidence interval (whisker). For the baseline survey, only the average number of contacts is indicated (dashed line). The baseline survey was conducted in the period from February 2016 to October 2017. Note that in the upper panel, the population is divided in two medical risk strata (high and low).

### Transmission potential

**Figure 5:**
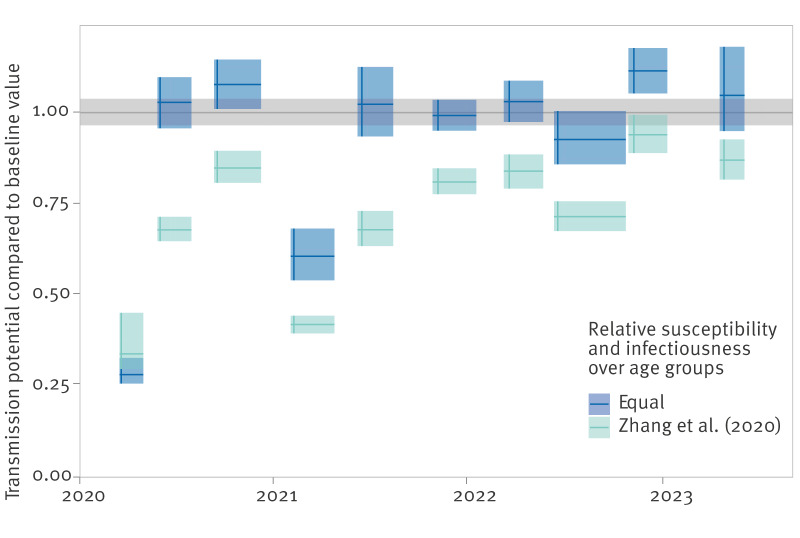
Transmission potential compared with the baseline value, the Netherlands, April 2020–November 2022 (n = 62,103)

To study the impact of the post-pandemic contact behaviour on the transmission potential of a new respiratory pathogen, we summed the contact matrices of the community and household contacts and assumed values for the relative susceptibility and infectiousness per age group. When all age groups are equally susceptible and infectious, the spectral radius of the resulting matrix is around the baseline value during most of the study period, except for survey rounds 1 and 4 when measures were most stringent (Figure 5). When all age groups are equally susceptible, transmission is driven by the age groups with most contacts, which are the youngest age groups that exhibited contact behaviour similar to baseline during most of the PiCo survey rounds. When younger age groups are less susceptible according to Zhang et al. [2], the transmission potential is always lower than the baseline value except in survey round 1, as transmission becomes more driven by adult age groups that structurally decreased their number of contacts. Supplementary Figure S3 shows that other reported patterns of the relative susceptibility and infectiousness of COVID-19 lead to similar results.

Shown is the ratio (horizontal line) and 95% confidence interval (shaded area). The 10 PIENTER Corona study (PiCo) rounds are shown from the start to end date, with the median survey date (vertical line). The baseline value is shown in grey. The baseline survey was conducted in the period from February 2016 to October 2017.

The relative susceptibility and infectiousness over age groups are either equal, or they follow a susceptibility profile for the early phase of COVID-19: Zhang et al. [2] reported that 0–14-year-olds are 2.9 times less susceptible than adults aged 15–64 years and that adults aged 65 years and older are 47% more susceptible than adults aged 15–64 years.

## Discussion

In this study, we evaluated whether the Dutch general population reverted to its pre-pandemic contact behaviour after lifting COVID-19 measures. Our results show that the population average of community contacts had decreased by 13% in the survey rounds in November 2022 and May 2023 when all control measures had been lifted, compared with the baseline value in 2016–17. However, children did return to having a high number of contacts as before the pandemic, retaining their role as potential epidemic drivers. As a consequence, the transmission potential of a newly emerging infection that would spread by a respiratory route mainly depends on how susceptible and/or infectious children would be in the context of a new pathogen.

We found that, for children under 20 and adults over 60 years, the number of contacts have reverted to their baseline levels. Only adults in the 20–59-year age groups seem to consistently have fewer contacts. A possible explanation is that, as these age groups are more likely to be employed, they may have worked more hours from home [34] thereby having fewer contacts while working. This trend is further substantiated by the results of the survey question whether participants worked from home in the last week. We found that for all working participants in age groups 20–69 years, the fraction that did not go to their workplace for work approximately doubled in the last two survey rounds, compared with a baseline value (data provided in Supplementary Figure S4). This is in line with a different survey that reported 37% of employees worked partly from home in 2019, increasing to 45% in 2022 [35].

We found that, in the baseline survey, the average number of contacts per participant increased with education level. This difference was smaller or even reversed in the periods with lockdown measures (survey rounds 1 and 4), which was also observed in a contact study in Hungary [36]. An explanation is that participants with a lower education level more often have work in essential sectors that preclude working from home [16]. This is confirmed by our results from the survey question whether participants worked from home in the last week stratified by education level. During the lockdown periods, around 70% of the highly educated participants worked (partly) from home, against 30% of the participants with a low education level. In the last two survey rounds, these percentages dropped to 50% and 25% respectively, which is still higher than the baseline values of 30% and 10%, respectively.

We found that the number of contacts did not differ between medical risk groups (i.e. indication for influenza vaccination) in any of the survey rounds nor the baseline survey. This was confirmed by the COVIMOD contact study in Germany [14] that used the same medical risk definition. However, in some study rounds of the CONNECT study in Canada, it was found that participants with comorbidities had fewer contacts than participants without comorbidities [13]. This would suggest that the absence of an association between number of contacts and medical risk status could be caused by our broad risk definition; persons with a very high medical risk may well have reduced their number of contacts. Participants with a high medical risk could also have had safer contacts with reduced risk of transmission by keeping distance and/or wearing protection. However, for these types of contacts, we found no difference between medical risk groups in our study (data provided in Supplementary Figure S5).

Our finding that the different contact patterns have a limited impact on the spread of a new pathogen if all age groups are equally susceptible is in contrast with the findings reported in the final round of another contact study (CoMix) held in November 2022 in four European countries [37]. For the Netherlands, an average number of 9.9 (9.0–10.8) contacts per person per day was found, which is lower than the 15.4 (14.5–16.2) contacts per person per day that we found in November 2022 (PiCo survey round 9). Using an earlier survey from 2006 to 2007 as baseline [38], the CoMix study reported that the transmission potential in the Netherlands had decreased by 20% in November 2022, while our study suggests no change in transmission potential when assuming equal susceptibility and infectiousness over age groups. Possible explanations could be found in the different design, study population and study period of the baseline survey, and in the fatigue effect observed for the Dutch data from the CoMix study which showed that participants report decreasing numbers of contacts because of the high frequency of reporting [6,39].

The strengths of this study include the identical study design of the baseline and PiCo surveys, which enabled the comparison of current to past contact behaviour. Both surveys were conducted with participants of all age groups, which is essential to study the spread in the entire population. The CONNECT study in Canada has a similar study design [9]; a post-pandemic survey of this study would be valuable to which we could compare our main results. Another strength is that the contact questionnaire is embedded in a larger serosurveillance study, providing a wealth of additional information on the participants. This allowed us to stratify our analysis by medical risk status and education level.

Our study has some limitations. Firstly, the possibility exists that the study population was on average more compliant to COVID-19 measures than the general population. Evidence for this is for instance reflected by their higher COVID-19 vaccine uptake [22]. Secondly, participants may have reported contacts differently compared with the baseline survey, because they have been made more aware of their contacts by COVID-19 information campaigns. Finally, we have weighted participants by age and sex, but not by education level because of the lack of reference values for participants younger than 15 years. Since participants with a high education level are overrepresented and tend to have a higher number of contacts than average with relaxed measures and a lower number of contacts than average with stringent measures, a consequence could be that the numbers of contacts are overestimated in periods with relaxed measures and underestimated in periods with stringent measures.

## Conclusions

Contact surveys have proven valuable during the COVID-19 pandemic in monitoring changes in contact behaviour with evolving measures and compliance. The baseline and PiCo surveys have provided direct quantitative evidence to inform tailored infection control policies in the Netherlands. Continuation of such contact surveys is therefore essential, even in a non-pandemic setting, not only to signal changes in contact patterns that affect the transmission potential, but also to provide a baseline for the next pandemic.

## Supplementary Data

1376000 Supplement
