# Supplementary material for: Contact behaviour before, during and after the COVID-19 pandemic in the Netherlands: evidence from contact surveys, 2016 to 2017 and 2020 to 2023
Source: Euro Surveill. 2024 Oct 24;29(43):2400143. doi: 10.2807/1560-7917.ES.2024.29.43.2400143 (PMC11513762; doi:10.2807/1560-7917.ES.2024.29.43.2400143)
Supplement: Supplement [file 24-00143_BACKER_Supplement.pdf]

This supplementary material is hosted by Eurosurveillance as supporting information alongside the article “Contact behaviour before, during and after the COVID-19 pandemic in the Netherlands: evidence from contact surveys, 2016 to 2017 and 2020 to 2023”, on behalf of the authors, who remain responsible for the accuracy and appropriateness of the content. The same standards for ethics, copyright, attributions and permissions as for the article apply. Supplements are not edited by Eurosurveillance and the journal is not responsible for the maintenance of any links or email addresses provided therein.

Supplementary material to ‘’Contact behaviour before, during and after the COVID-19 pandemic in the Netherlands: evidence from contact surveys, 2016 to 2017 and 2020 to 2023’’

Jantien A. Backer[1], Eric R. A. Vos[1], Gerco den Hartog[1], Cheyenne C. E. van Hagen[1], Hester E. de Melker[1], Fiona R. M. van der Klis[1], Jacco Wallinga[1,2]

[1] National Institute for Public Health and the Environment (RIVM), Bilthoven, the Netherlands

[2] Leiden University Medical Center, Leiden, the Netherlands

S1 Study design and data

S2 Study population weighted by age group and sex

S3 Sensitivity analysis for transmission potential

S4 Working from home

S5 Contact type by medical risk group

S1 Study design and data

Details on the study design and selection of participants were described in publications on the baseline survey [1] and the PiCo surveys [2–4]. Here we will briefly summarise aspects that are relevant to our study.

Participant selection

For the baseline survey, participants were randomly selected from the national Personal Records Database (BRP) [5] via a two-stage cluster design, comprising 40 municipalities in five regions nationwide [1]. The national sample consisted of 5745 participants (response rate of 14.4%). The participants who consented to be approached for follow-up and could be contacted (i.e. 80% of the baseline survey participants) were invited for the first round of the PiCo survey in April 2020, 53% of whom participated [2]. For the second PiCo round (June 2020), 26,854 additional participants were randomly selected from the BRP from all regions of the Netherlands, 16.7% of whom participated [3]. The participants of PiCo rounds 1 and 2 were reinvited for all subsequent rounds. For the sixth PiCo round (November 2021), 65,690 additional participants were randomly selected from the BRP from all regions of the Netherlands, 4.8% of whom participated [4]. Also these new participants of PiCo rounds 6 were reinvited for all subsequent rounds. The participation rate for each of these groups of participants is shown in Table **S1-1**. Note that these are lower bounds as participants may have requested to be removed from the study or may have passed away over the course of the survey. In Table **S1-2** the median, start and end date for each round are listed. These dates are used in Figures 2, 3, and 5 in the main text.

Table S1-1: Participation by groups (G) that were invited for PiCo rounds 1, 2 and 6, and overall participation. Columns show the number of participants (n) and participation rates (%).

Round	G1 (n)	G1 (%)	G2 (n)	G2 (%)	G6 (n)	G6 (%)	Overall (n)	Overall (%)
1	2,594	100.0					2,594	100.0
2	2,207	85.1	4,497	97.2			6,704	92.8
3	2,037	78.5	4,049	87.5			6,086	84.3
4	2,036	78.5	3,876	83.8			5,912	81.9
5	1,824	70.3	3,407	73.6			5,231	72.4
6	1,691	65.2	3,167	68.4	3,286	98.9	8,144	77.2
7	1,355	52.2	2,527	54.6	2,465	74.2	6,347	60.2
8	1,234	47.6	2,307	49.8	2,085	62.7	5,626	53.4
9	1,193	46.0	2,187	47.3	1,868	56.2	5,248	49.8
10	1,126	43.4	1,992	43.0	1,712	51.5	4,830	45.8

Table S1-2: Time frame of survey rounds of baseline survey (round 0) and PiCo survey rounds. Shown are median, start and end date for each round.

Round	Median date	Start date	End date
0	2017-01-29	2016-01-10	2017-12-03
1	2020-04-02	2020-03-30	2020-05-13
2	2020-06-13	2020-06-08	2020-08-10
3	2020-09-27	2020-09-21	2020-12-17
4	2021-02-16	2021-02-10	2021-05-02
5	2021-06-22	2021-06-14	2021-08-19
6	2021-11-10	2021-10-31	2022-01-20
7	2022-03-22	2022-03-12	2022-05-24
8	2022-06-20	2022-06-13	2022-10-24
9	2022-11-08	2022-10-29	2023-01-09
10	2023-04-24	2023-04-16	2023-06-01

Changes in survey questions

Most questions on participant and contact characteristics remained the same over the course of all rounds, including the baseline survey. Some survey questions however changed during the study period, summarised in Table **S1-3**.

Contacts' ages were reported in age groups: 0–4, 5–9, 10–19, 20–29, 30–39, 40–49, 50–59, 60–69, 70–79, 80–89 and ≥ 90, but from PiCo round 3 onwards the contact age group 10-19 was subdivided in 10-14 and 15-19. Participants could further distinguish their contacts, but this distinction changed from round to round. In the baseline survey and Pico rounds 1 and 8, the number of men and women in each contact age group was reported. In PiCo rounds 2, 3, and 10 this distinction was replaced by whether contacts took place within or outside 1.5 meters. In all other PiCo rounds, the category for contacts within 1.5 meters, was further subdivided in whether the contact occurred with or without personal protective equipment, such as a face mask.

A question about working from home was included in the PiCo survey. To obtain a baseline value, participants of rounds 1 and 2 were also asked about working from home in the period preceding the COVID-19 pandemic.

Table S1-3: Survey questions that have changed during the study period of the baseline survey (indicated as round 0) and the PiCo survey (rounds 1 to 10). The close/distant distinction is defined as less/more than 1.5 m apart. An 'x' denotes which version of the survey question was posed in that round.

Survey question		0	1	2	3	4	5	6	7	8	9	10
Household composition	including participant	x	x									
	excluding participant			x	x	x	x	x	x	x	x	x
Medical conditions	currently or in past	x	x	x								
	currently					x	x	x	x	x	x	x
Working from home	before COVID-19		x	x								
	last week		x	x	x	x	x	x	x	x	x	x
Check on contacts			x	x	x	x	x	x	x	x	x	x
Contact age group 10-19	10-19	x	x	x								
	10-14 and 15-19				x	x	x	x	x	x	x	x
Contact distinction	men / women	x	x							x		
	close / distant			x	x							x
	close with/out protection / distant					x	x	x	x		x	

Contact question

The contact question as was asked in PiCo round 7 is shown in Figure **S1-1**. The check on the number of contacts (Did you have contacts with other persons yesterday?) was only present in the Pico surveys, not in the baseline survey. The distinctions to be made between contacts could differ by PiCo round (see Tab. **S1-3**).

10. How many persons did you have contact with yesterday (excluding household members)?

A contact happens when you, for instance, talk to someone, touch someone, kiss someone or play sports with someone. Also remember contacts at work or school. Telephone conversations, e-mail and other online contacts should not be counted.

Please distinguish between ***protected*** *and* ***unprotected*** *contacts that happened within 1.5 meters and contacts that happened at a distance of 1.5 m or more. Protected contacts are, for instance, when both persons (you and the person you have contact with) wear a face mask or face shield or when a plastic barrier is placed between both persons (such as at the cashier in shops).*

Indicate this by age group.

This is a difficult question; we'd like to emphasize that an approximate answer suffices.

Did you have contacts with other persons yesterday (excluding household members)?

☐ Yes, fill out the table below (you can leave cells empty)

☐ No → **continue with question 11**

NB Indicate in NUMBERS how many contacts you had in the tabel below. Do not use crosses or other symbols to indicate the number of contacts.

**Age group**	**Number of unprotected contacts WITHIN 1,5 meters**	**Number of protected contacts WITHIN 1,5 meters**	**Number of contacts at a distance of 1.5 meters or more**
0 - 4 year			
5 - 9 year			
10 - 14 year			
15 - 19 year			
20 - 29 year			
30 - 39 year			
40 - 49 year			
50 - 59 year			
60 - 69 year			
70 - 79 year			
80 - 89 year			
90+ year			

11. Yesterday was a:

☐ Monday ☐ Tuesday ☐ Wednesday ☐ Thursday ☐ Friday ☐ Saturday ☐ Sunday

Figure S1-1: Contact question of PiCo round 7 (translated from Dutch).

Valid household composition

Participants were asked about their household size and composition, i.e. the age and sex of all household members. In the baseline survey and round 1 of the PiCo survey, the participants should include themselves, but from round 2 of the PiCo survey they should exclude themselves. As a consequence, from round 2 onwards the household composition could be empty, either because the participant lived in a single-person household or because they skipped the question. All non-empty household compositions were indicated to be valid, as well as empty household compositions of participants who reported to live in a single-person household from round 2 onwards. To harmonize the different surveys, persons with the same age and sex as the participant were deleted from the reported household composition in the baseline survey and round 1 of the PiCo survey. Participants without a valid household composition are only excluded for the analysis of contacts with household members, but they are included for the analysis of community contacts.

Medical risk status

In the baseline survey and PiCo rounds 1 and 2, the participant was asked about current and previous medical conditions. From PiCo round 4 onwards, the question was restricted to current medical conditions. In PiCo round 3 no medical questions were posed.

Using these medical conditions, the medical risk status of each participant was based on whether they would be indicated for influenza vaccination. Following the current guidelines [6] these conditions include diabetes (any kind), respiratory disease, liver disease, immunocompromised condition, cancer, asplenia, renal disease, cardiovascular disease, neurological condition, transplant patients, and/or morbid obesity (Body Mass Index ≥ 40, calculated from the length and weight of the participant). When at least one of these conditions applied, the participant was classified in the high medical risk category. When none of the medical condition questions were answered (or posed), the medical risk status was imputed. Because the medical risk questions changed around PiCo round 3, we imputed the missing medical risk status for the periods before round 3 and from round 3 onwards separately. For each period, it was determined whether a participant had an unambiguous risk status, i.e., only high or only low, apart from the missing data. If so, the missing risk status was replaced by the unambiguous risk status. For instance, a participant with a medical risk status of high, missing and missing in round 0, 1 and 2 would have an unambiguous high risk status, and the missing risk status would be replaced by high medical risk. On the other hand, a participant with a medical risk status of high, low and missing in round 0, 1 and 2 would have an ambiguous risk status, and the missing risk status is not imputed.

Valid contact data

Only participants who provided valid contact data are included in the data. In the PiCo survey, participants were first asked whether they had had any contacts outside the household on the previous day, before filling out the actual number of contacts. Participants who answered 'yes' and reported a number of contacts, and participants who answered 'no' and reported zero

contacts were included. Participants who answered ‘yes’ but did not report any contacts, and participants who answered ‘no’ but did report a number of contacts were excluded. Participants who did not answer the check question were only included if they provided a number of contacts. Participants who did not answer the check question and did not provide any contacts were excluded, presuming they skipped the question.

The baseline survey lacked the question to check whether a participant had any contacts on the previous day. Instead, we used the question on which day it was yesterday (question 11 in **1**) as a check. Participants who filled out this questions were included, with and without reported contacts. Participants who did not provide the contact day were only included if they reported a number of contacts. Participants who did not provide the contact day and did not report any contacts were excluded, presuming they skipped the question.

S2 Study population corrected for confounding by age group and sex

Table S2: Study population characteristics corrected for confounding, using the age group and sex of the general population [5]. The survey month, the number of participants per survey round and stratified in percentages by household size, medical risk group and education level. The baseline survey from 2016-2017 is indicated as survey round 0. Before weighting the missing values were omitted. The final column contains reference percentages for the household size [7], medical risk group [8], and education level for 15-90 year olds [6].

round		0	1	2	3	4	5	6	7	8	9	10	ref
Survey month			Apr 2020	Jun 2020	Oct 2020	Mar 2021	Jul 2021	Nov 2021	Apr 2022	Jul 2022	Nov 2022	May 2023	
Number of participants		5381	2594	6704	6086	5912	5231	8144	6347	5626	5248	4830	
Household size	1	22.0	13.5	11.3	11.0	11.2	11.4	11.9	12.1	13.0	11.9	12.2	18.1
	2	34.9	31.6	33.3	33.9	34.2	34.4	34.6	35.1	35.6	35.1	35.2	30.7
	3	12.4	15.6	14.6	14.6	14.1	13.6	13.8	13.4	13.1	13.3	13.4	16.6
	4	19.5	26.9	26.6	26.1	26.3	26.9	25.8	25.9	24.9	25.4	24.8	22.6
	5+	11.2	12.5	14.2	14.4	14.2	13.7	13.9	13.5	13.4	14.2	14.3	12.0
Medical risk group	Low	71.1	69.3	70.7	81.7	79.5	80.7	77.4	79.5	79.9	79.3	78.4	78.6
	High	28.9	30.7	29.3	18.3	20.5	19.3	22.6	20.5	20.1	20.7	21.6	21.4
Education level	Low	30.8	22.6	20.3	19.4	18.6	18.2	18.0	16.9	17.0	16.7	16.8	28.6
	Medium	33.5	34.4	32.3	32.9	32.9	33.1	31.8	31.6	31.5	31.8	32.2	37.8
	High	35.7	43.0	47.4	47.7	48.5	48.7	50.3	51.5	51.6	51.5	51.1	33.6

S3 Sensitivity analysis for transmission potential

To assess how sensitive the transmission potential is to assumptions on the relative susceptibility and infectiousness by age group for COVID-19, we tried several parameterisations from literature. Zhang et al. [7] estimated susceptibility by age group, while keeping infectiousness constant. Franco et al. [8] used an NGM approach fixing either infectiousness and estimating susceptibility (scenario A) or vice versa (scenario B). Finally, Klinkenberg et al. [9] assumed susceptibility and infectiousness were varying by age group but identical. Although these assumptions lead to slightly different estimates for the transmission potential, they all are distinctly different from the assumption that susceptibility and infectiousness are equal for all age groups (Fig. **S3**).

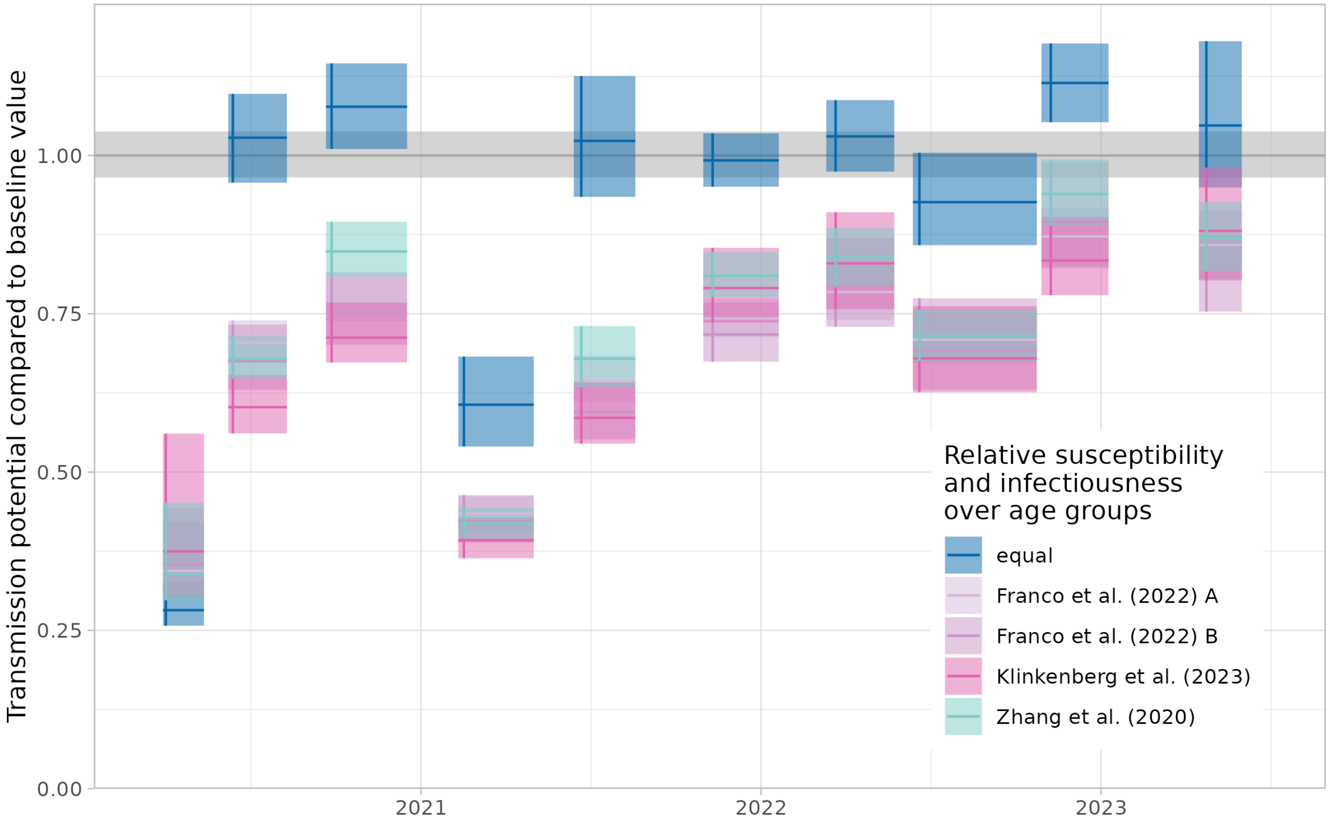

Figure S3: Transmission potential, expressed as the spectral radius of the next generation matrix (NGM) compared to the baseline value, for different assumptions of relative susceptibility and infectiousness over age groups. Shown is the ratio (horizontal line) and 95% bias-corrected bootstrap interval (shaded area). PiCo rounds are shown from the start to end date, with the median survey date (vertical line).

Table S3: Assumptions for relative susceptibility and infectiousness by age group, for sensitivity analysis of transmission potential.

reference	type	0-4	5-9	10-19	20-29	30-39	40-49	50-59	60-69	70-79	80+
equal	sus	1.000	1.000	1.000	1.00	1.000	1.000	1.00	1.000	1.000	1.000
	inf	1.000	1.000	1.000	1.00	1.000	1.000	1.00	1.000	1.000	1.000
Franco et al. (2022) A	sus	0.182	0.550	0.603	1.00	1.172	1.009	0.88	0.869	0.846	0.805
	inf	0.540	0.550	0.560	0.59	0.700	0.760	0.90	0.990	0.990	0.990
Franco et al. (2022) B	sus	0.400	0.390	0.380	0.79	0.860	0.800	0.82	0.880	0.740	0.740
	inf	0.346	0.892	1.310	1.00	0.645	3.783	1.32	0.266	1.277	0.099
Klinkenberg et al. (2023)	sus	1.000	1.000	3.050	5.75	3.540	3.710	4.36	5.690	5.320	7.210
	inf	1.000	1.000	3.050	5.75	3.540	3.710	4.36	5.690	5.320	7.210
Zhang et al. (2020)	sus	0.340	0.340	0.670	1.00	1.000	1.000	1.00	1.235	1.470	1.470
	inf	1.000	1.000	1.000	1.00	1.000	1.000	1.00	1.000	1.000	1.000

S4 Working from home

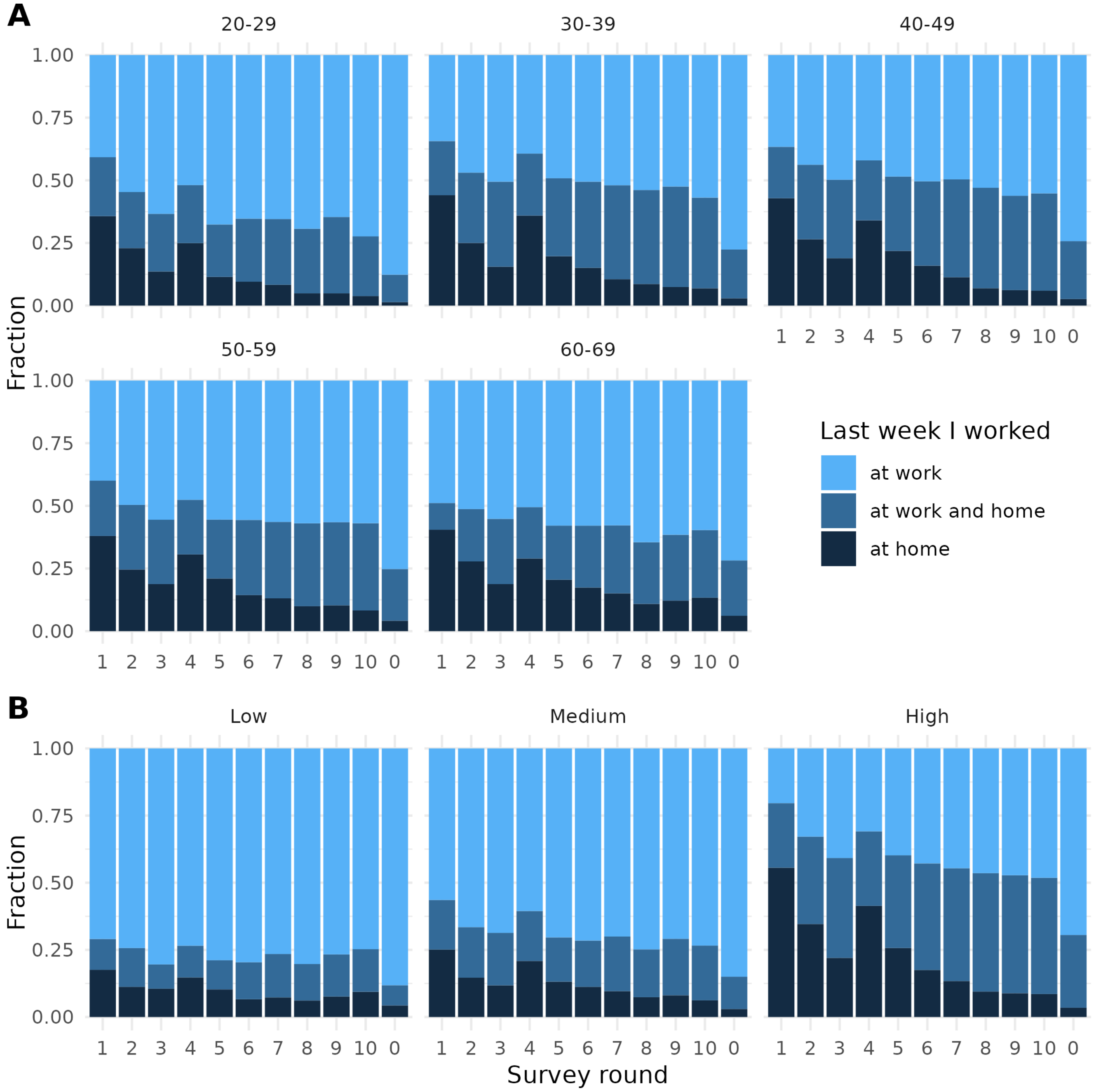

Figure S4: Fraction of participants with employment in working ages 20-69 that (partly) worked from home in the previous week, by (A) age group and (B) education level. The baseline value (at round 0, for ease of comparison plotted next to the most recent rounds) is the fraction of participants who (partly) worked from home in the pre-COVID-19 period, answered by participants in rounds 1 and 2.

S5 Contact type by medical risk group

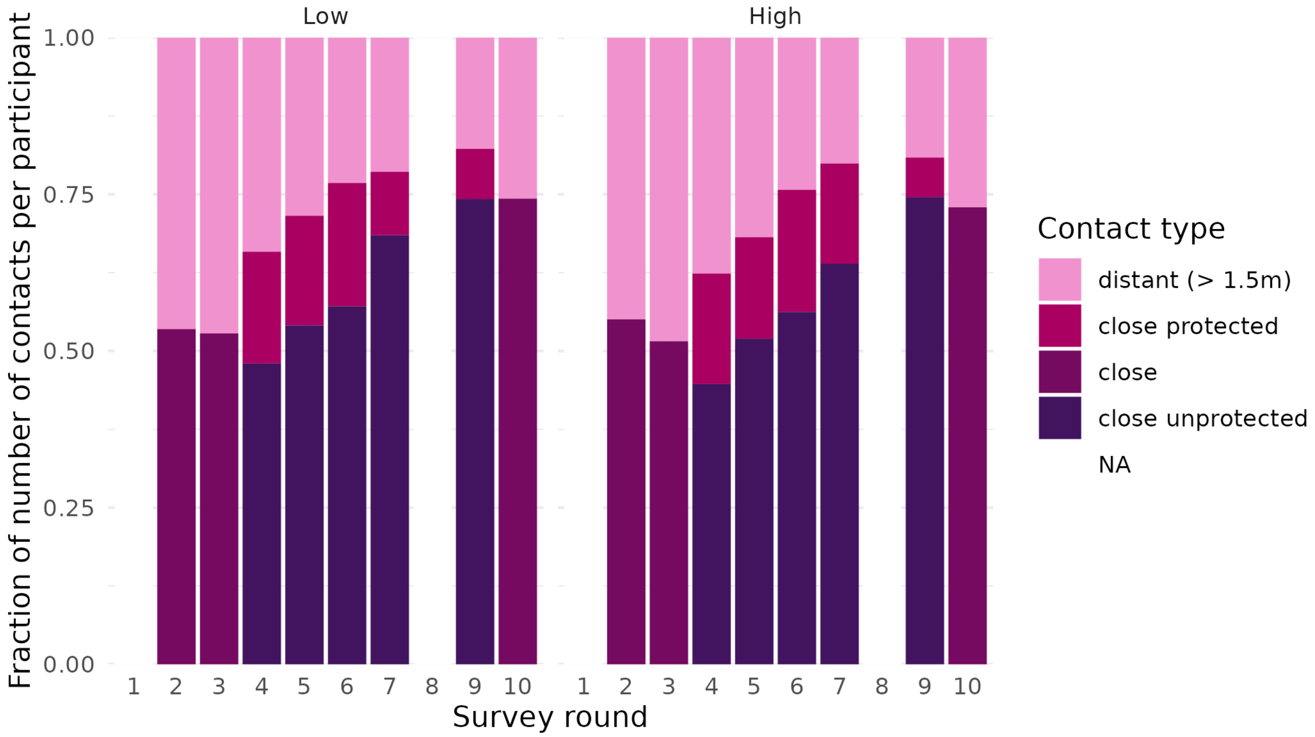

Figure S5: Fraction of contacts per participant, stratified by contact type: distant (more than 1.5 m), close (less than 1.5 m), close unprotected (less than 1.5 m without protection), close protected (less than 1.5 m with protection) by survey round. In rounds 1 and 8 contacts were stratified by gender. Fractions are weighted by age group distribution of the general population.

References

1. Verberk JDM, Vos RA, Mollema L, van Vliet J, van Weert JWM, de Melker HE, et al. Third national biobank for population-based seroprevalence studies in the Netherlands, including the Caribbean Netherlands. BMC Infect Dis. 2019;19: 470. doi:10.1186/s12879-019-4019-y

2. Vos ERA, Hartog G den, Schepp RM, Kaaijk P, Vliet J van, Helm K, et al. Nationwide seroprevalence of SARS-CoV-2 and identification of risk factors in the general population of the Netherlands during the first epidemic wave. J Epidemiol Community Health. 2020. doi:10.1136/jech-2020-215678

3. Vos ERA, Boven M van, Hartog G den, Backer JA, Klinkenberg D, Hagen CCE van, et al. Associations Between Measures of Social Distancing and Severe Acute Respiratory Syndrome Coronavirus 2 Seropositivity: A Nationwide Population-based Study in the Netherlands. Clin Infect Dis. 2021;73: 2318–2321. doi:10.1093/cid/ciab264

4. Vos ERA, Hagen CCE van, Wong D, Smits G, Kuijer M, Wijmenga-Monsuur AJ, et al. SARS-CoV-2 seroprevalence trends in the Netherlands in the variant of concern era: Input for future response. Influenza Other Respi Viruses. 2024;18: e13312. doi:10.1111/irv.13312

5. Government of the Netherlands. Personal Records Database (BRP). 2023. Available: https://www.government.nl/topics/personal-data/personal-records-database-brp

6. National Institute for Public Health and the Environment (RIVM). Influenzavaccinatie [Influenza vaccination], updated 3 May 2023 (accessed 29 January 2024). Bilthoven: RIVM; 2023. Available: https://lci.rivm.nl/richtlijnen/influenzavaccinatie

7. Zhang J, Litvinova M, Liang Y, Wang Y, Wang W, Zhao S, et al. Changes in contact patterns shape the dynamics of the COVID-19 outbreak in China. Science. 2020;368: 1481–1486. doi:10.1126/science.abb8001

8. Franco N, Coletti P, Willem L, Angeli L, Lajot A, Abrams S, et al. Inferring age-specific differences in susceptibility to and infectiousness upon SARS-CoV-2 infection based on Belgian social contact data. PLoS Comput Biol. 2022;18: e1009965. doi:10.1371/journal.pcbi.1009965

9. Klinkenberg D, Backer J, Keizer N de, Wallinga J. Projecting COVID-19 intensive care admissions for policy advice, the Netherlands, February 2020 to January 2021. Euro Surveill. 2024;29. doi:10.2807/1560-7917.ES.2024.29.10.2300336

10. Statistics Netherlands. Bevolking op eerste vd maand; geslacht, lft, migratieachtergrond; 2016-2023 [Population on the first of the month; by sex, and age, migration background; 2016-2023]. (Accessed 8 August 2023). The Hague: CBS; 2023. Available: https://opendata.cbs.nl/#/CBS/nl/dataset/83482NED/table?dl=98643

11. Statistics Netherlands. Bevolking; hoogstbehaald onderwijsniveau en onderwijsrichting, 2003-2022 [Population; highest level of education and field of study,

2003-2022]. (Accessed 10 October 2023). The Hague: CBS; 2023. Available: https://opendata.cbs.nl/statline/#/CBS/nl/dataset/85184NED/table?dl=984E1

12. Statistics Netherlands. Particuliere huishoudens naar samenstelling en grootte, 1 januari [Private households by composition and size, 1 January. (Accessed 29 February 2024). The Hague: CBS; 2024. Available: https://opendata.cbs.nl/statline/#/CBS/nl/dataset/37975/table?dl=9E579

13. Heins MJ, Korevaar JC, Knottnerus BJ, Hooiveld M. Vaccine Coverage Dutch National Influenza Prevention Program 2021: brief monitor. (Accessed 29 January 2023). Utrecht: Nivel; 2022. Available: https://www.nivel.nl/sites/default/files/bestanden/1004308.pdf
